# Heat stress combined with lipopolysaccharide induces pulmonary microvascular endothelial cell glycocalyx inflammatory damage in vitro

**DOI:** 10.1002/iid3.1034

**Published:** 2023-10-05

**Authors:** Jiadi Chen, Chengjia Ding, Jingjing Cao, Huasheng Tong, Yi Chen

**Affiliations:** ^1^ Department of Intensive Care Medicine First Ward The First Affiliated Hospital of Shantou University Medical College Shantou Guangdong China; ^2^ Department of Critical Care Medicine, Binhaiwan Central Hospital of Dongguan Dongguan Hospital Affiliated to Jinan University Dongguan Guangdong China; ^3^ The Key Laboratory for Prevention and Treatment of Critical Illness in Dongguan City Dongguan Guangdong China; ^4^ Department of Emergency Medicine General Hospital of Southern Theatre Command of PLA Guangzhou Guangdong China

**Keywords:** glycocalyx, heat stroke, human pulmonary microvascular endothelial cells

## Abstract

Heat stroke is a life‐threatening disease with high mortality and complications. Endothelial glycocalyx (EGCX) is essential for maintaining endothelial cell structure and function as well as preventing the adhesion of inflammatory cells. Potential relationship that underlies the imbalance in inflammation and coagulation remains elusive. Moreover, the role of EGCX in heat stroke‐induced organ injury remained unclear. Therefore, the current study aimed to illustrate if EGCX aggravates apoptosis, inflammation, and oxidative damage in human pulmonary microvascular endothelial cells (HPMEC). Heat stress and lipopolysaccharide (LPS) were employed to construct in vitro models to study the changes of glycocalyx structure and function, as well as levels of heparansulfate proteoglycan (HSPG), syndecan‐1 (SDC‐1), heparansulfate (HS), tumor necrosis factor‐α (TNF‐α), interleukin (IL)‐6, Von Willebrand factor (vWF), endothelin‐1 (ET‐1), occludin, E‐selectin, vascular cell adhesion molecule‐1 (VCAM‐1), and reactive oxygen species (ROS). Here, we showed that heat stress and LPS devastated EGCX structure, activated EGCX degradation, and triggered oxidative damage and apoptosis in HPMEC. Stimulation of heat stress and LPS decreased expression of HSPG, increased levels of SDC‐1 and HS in culture supernatant, promoted the production and release of proinflammation cytokines (TNF‐α and IL‐6,) and coagulative factors (vWF and ET‐1) in HPMEC. Furthermore, Expressions of E‐selection, VCAM‐1, and ROS were upregulated, while that of occludin was downregulated. These changes could be deteriorated by heparanase, whereas they meliorated by unfractionated heparin. This study indicated that EGCX may contribute to apoptosis and heat stroke‐induced coagulopathy, and these effects may have been due to the decrease in the shedding of EGCX.

## Graphical abstract

The features of vascular endothelial glycocalyx under stimulation of heat stress and lipopolysaccharide corroborated vascular inflammatory injury and coagulation disorder. For the first time, the study showed that heat stroke triggered endothelial glycocalyx degradation and further induced oxidative damage and apoptosis of human pulmonary microvascular endothelial cells, which impaired its ability to resist inflammatory injury and maintaining vascular permeability.

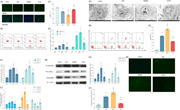

## 1 INTRODUCTION

Heat stroke is identified as multiorgan dysfunction syndrome (MODS) secondary to systemic inflammatory response (SIRS) triggered by hyperthermia,^1^, ^2^ with mortality rate reached 63.2% even though under intensive care.^3^ Heat cytotoxicity, coagulopathies, and systemic inflammation induced by vascular endothelium were believed as the critical mechanism of MODS.^4^, ^5^ The structural and functional stability of vascular endothelium was maintained by endothelial glycocalyx (EGCX), which prevents the adhesion of inflammatory cells.^6^

Kobayashi et al.^7^ have been reported that heat stress decreased the thickness of the cardiac capillary EGCX, indicating that heat stroke contributed to the degradation of EGCX.^8^ Shedding of EGCX‐induced vascular endothelial dysfunction magnifies the inflammatory response in sepsis and promotes SIRS to MODS.^9^, ^10^ Microcirculation was damaged, and vascular permeability was increased by the degradation of EGCX in systemic inflammation.^11^, ^12^, ^13^ Taken together, we hypothesize that degradation of EGCX may also play critical roles in inflammation and coagulation during heat stroke.

The key motivation of inflammation‐coagulation reaction induced by heat stroke has been suggested to be associated with enterogenous endotoxemia. Dislocation of endotoxin was observed secondary to the injury of intestinal mucasa, which increased the level of endotoxin in the blood.^14^ Intestinal lesions in mice worsened with the increase in rectal core temperature during heat stress.^15^ There was a synergistic effect between heat stress and damaged intestinal lesions‐induced translocation of bacterial endotoxin, which was believed as a key link in the pathogenesis of severe heat stroke. Hyperthermia may facilitate the early leakage of lipopolysaccharide (LPS) from the intestine to the systemic circulation, which excessively activates endothelial cells to exaggerate the inflammatory and coagulation responses.^1^

Collectively, the present study aims to confirm the effects of degradation of EGCX in heat stroke‐induced coagulopathy. Human pulmonary microvascular endothelial cell (HPMEC) was explored to observe the injury characteristic of vascular EGCX under heat stress combined with LPS double hits in *vitro* models.

## 2 MATERIALS AND METHODS

### 2.1 Preparation of HPMEC

HPMEC was purchased from Guangzhou Dewei Biological Technology Co. Ltd, China. HPMEC was cultured in the endothelial cell medium with 10% fetal bovine serum (ScienCell) at 37°C in a humidified incubator with 5% CO_2._ All experiments were carried out during the logarithmic phase of cell growth. The account of cells was adjusted to 1 × 10^6^/mL by serum‐free medium before culture, inoculating into six‐well plates, and incubated at 5% CO_2_ and 37°C.

### 2.2 Induction of the HPMEC under heat stress combined with LPS in vitro

#### 2.2.1 Pretreatment before modeling

Heat stress and lipopolysaccharide (HS + LPS) were employed to construct the in vitro models of heat stroke in our previous study.^16^ All the HPMEC were categorized into four groups with eight wells in each group: control group (CON group), heat stroke group (HS group), heparanase group (HPSE group), and unfractionated heparin group (UFH group). After conventional culture for 72 h, the cell in the CON group was cultured in normal conditions, without receiving heat stress or LPS (Sigma). No pretreatment was performed on the HS group before modeling. The cell in the HPSE group was subjected to a 0.2 U/mL Heparanase (Sigma) culture medium and incubated at 37°C for 30 min. The UFH group was subjected to a medium containing 10 U/mL unfractionated heparin (Sigma) and incubated 12 h before modeling.^17^, ^18^, ^19^

#### 2.2.2 Modeling

Briefly, except for the CON group, HPMEC in HS, HPSE, and UFH groups were subjected to heat stress at 43°C for 30 min, and then moved to 5% CO_2_, 37°C incubators, stimulated with LPS at a concentration of 500 ng/mL for 12 h.

### 2.3 Detection of changes in EGCX structure and function

#### 2.3.1 Flow cytometry

To investigate HPMEC apoptosis and heparansulfate proteoglycan (HSPG) concentration, flow cytometry was performed in each group according to our previous methods.^16^ Cells in each group were washed with phosphate‐buffered saline (PBS) for 15 min, centrifuged, and resuspended with 500 μL AnnexinV‐fluorescein isothiocyanate (FITC) (UElandy) binding buffer per tube. The cells were further stained with 5 μL AnnexinV‐FITC, then mixed and incubated in the dark at 4°C for 15 min. Lastly, 10 μL propidium iodide (PI) staining solution was added, and the flow cytometry (Becton, Dickinson and Company) was performed to detect after incubated in the dark for 5 min. Data compensation and analysis were performed using FlowJo version 7.6.1.

#### 2.3.2 Measurements of reactive oxygen species (ROS)

To examine the intracellular ROS levels, a ROS assay kit that sets dichloro‐dihydro‐fluorescein diacetate (DCFH‐DA) as the probe was used. HPMEC supernatant was withdrawn after modeling from each group, and DCFH‐DA was diluted to 10 μmol/L by 1:1000 in a serum‐free blank medium. Cells were stained with DCFH‐DA (Nanjing Jiancheng) in a 37°C cell incubator for 20 min. The cells were washed three times with a blank medium, observed and photographed by Olympus positive fluorescence system microscope (Olympus Corporation), and analyzed by ImageJ software.

#### 2.3.3 Immunofluorescent staining

Cells from each group were washed with PBS for three times (1 min each), washed in PBS for 5 min each. Cells were then permeabilized in precooled 0.25% Triton X‐100 solution for 10 min on ice, blocked in 1% bovine serum albumin (BSA; Sigma) for 1 h. Incubation with anti‐HSPG at 4°C for 2 h (Affinity Biosciences) was followed by three washes (5 min each) with PBS and incubation with Goat Anti‐rabbit complex at 1:20 dilution in the dark at room temperature for 1 h. Finally, the cells were incubated with 4′,6‐diamidino‐2‐phenylindole in the dark for 5 min and then were observed in a fluorescence microscope.

#### 2.3.4 Enzyme‐linked immunosorbent assay (ELISA) test

The level of major degraded products of EGCX, including syndecan‐1 (SDC‐1) and heparin sulfate (HS), was measured by ELISA based on an established procedure. Moreover, ELISA was also performed to detect the concentrations of tumor necrosis factor‐α (TNF‐α), interleukin‐6 (IL‐6), Von Willebrand factor (vWF), and endothelin‐1 (ET‐1).

#### 2.3.5 Western blot

The expression levels of E‐selectin, VCAM‐1, and Occludin in HPMEC were investigated via western blot. Cells were collected and lysed in radioimmunoprecipitation assay lysis buffer (LEAGENE), which added with phenylmethylsulfonyl fluoride for 30 min on ice. The protein samples were determined using BCA Protein Assay Kit (Cat#: 23227, Pierce), separated on 12% sulfate‐polyacrylamide gel electrophoresis, and were then transferred to polyvinylidene difluoride membranes (BIO‐RAD). The membranes were further blocked with 5% nonfat dry milk with 100 mL Tris‐buffered saline with 0.05% Tween 20 (TBST) at room temperature and incubated with rabbit anti‐E‐selectin (SAB), rabbit anti‐Occludin (SAB), rabbit anti‐VCAM1 (SAB) primary antibodies at 4°C overnight. TBST was used for rinsing for 10 min and shaking at room temperature. Peroxidase‐conjugated Affinipure goat anti‐rabbit IgG/FITC and goat anti‐mouse IgG/PE were used for incubating in the dark at room temperature for 1 h. The immunoblots were investigated by enhanced chemiluminescence (ECL), and the protein expression levels were analyzed by ImageJ software.

### 2.4 Detection of HPMEC ultrastructure

#### 2.4.1 Transmission electron microscopy (TEM)

The ultrastructure of HPMEC was assessed using TEM analysis. HPMEC were cultured on filter membranes were fixed in 2.5% glutaraldehyde in cacodylate buffer (PH 7.4) for 2 h, then fixed in 1% OsO4 (Sinopharm Chemical Reagent Co., Ltd.) for 2 h, dehydrated in a series of ethanol washes, and embedded in Epon 812 Resin according to the standard procedure. Ultrathin section (60–80 nm) was prepared, stained with both uranyl acetate and lead citrate and photographed using a Hitachi 7700 electron microscope.

### 2.5 Statistical analysis

All statistical analysis of the data was analyzed by SPSS 27.0 and GraphPad Prism 9. All the data were expressed as mean ± standard deviation (mean ± SD). If the variance is uniform, single‐factor descriptive analysis of variance (ANOVA) and least significant difference (LSD) methods were used to analyze comparisons among multiple groups. If not, the Welch test was used. *p* < .05 was considered to be statistically significant.

## 3 RESULTS

### 3.1 Heat stress and LPS‐induced damage and degradation of EGCX

#### 3.1.1 Glycocalyx content

**Figure 1:**
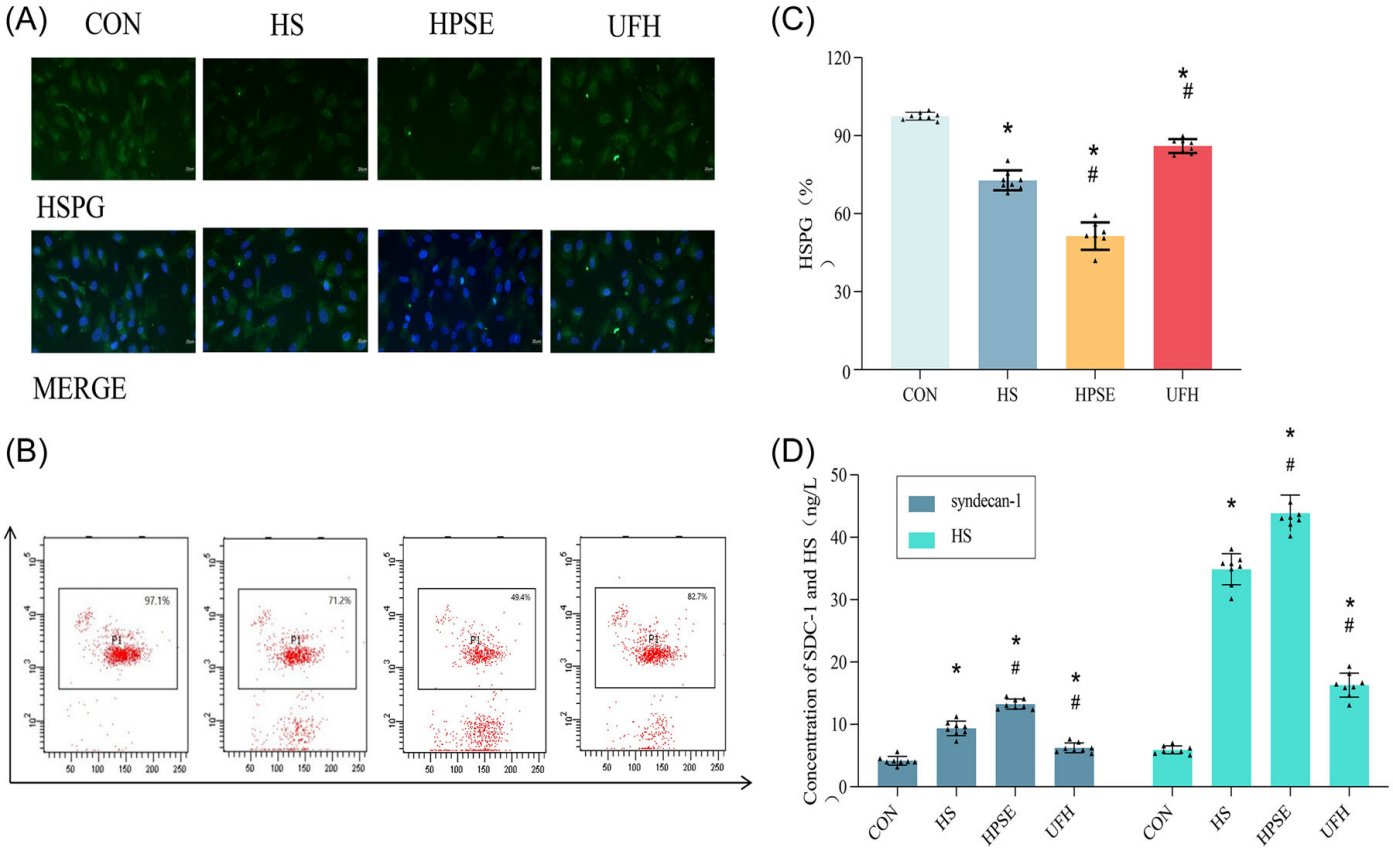
Heat stroke decreased the expression of HSPG and concentration of SDC‐1 and HS in HPMEC. (A). Representative immunofluorescence images of HSPG in glycocalyx from each group at magnification ×100. Green indicated HSPG, and blue indicated nuclear. (B and C). HSPG concentration in each group was detected using flow cytometry. (D). Concentration of SDC‐1 and HS in cell culture supernatant in HPMEC from each group. *p < .05 versus CON, ^#^ *p* < .05 versus HS; *n* = 8. CON, control; HPMEC, human pulmonary microvascular endothelial cells; HPSE, heparanase; HS, heparansulfate; HSPG, heparansulfate proteoglycan; SDC‐1, syndecan‐1; UFH, unfractionated heparin.

Given a previous study suggesting the importance of HSPG in accessing the content of EGCX,^20^ HSPG expression was first investigated in HPMEC among four groups under heat stress and LPS. Stimulation of heat stress and LPS decreased HSPG expression compared with those in the CON group (*p* < .05) (Figure 1A–C), as indicated by immunofluorescent staining and flow cytometry. Compared with the HS + LPS group, the HSPG expression was higher in the HPSE group while lower in the UFH group. (*p* < .05) (Figure 1A–C). These results indicated that heat stroke decreased the content of EGCX, which was attenuated by UFH.

#### 3.1.2 Levels of glycocalyx degradation products

Considering that SDC‐1 and heparin sulfate were identified as the classical degraded products of EGCX.^21^ The results suggested that the levels of SDC‐1 and heparinsulfate were significantly lower in the CON group than that in others (*p* < .05) (Figure 1D). UFH might prevent EGCX from degrading as evidenced by the lower expression in the UFH group compared with HS + LPS and HPSE groups (*p* < .05). These results revealed that heat stress and LPS may induce EGCX degradation in HPMEC, which showed a positive correlation with the degree of glycocalyx damage.

### 3.2 Heat stress and LPS triggered HPMEC apoptosis via activating EGCX degradation

#### 3.2.1 Changes in ultrastructure and apoptosis of HPMEC

**Figure 2:**
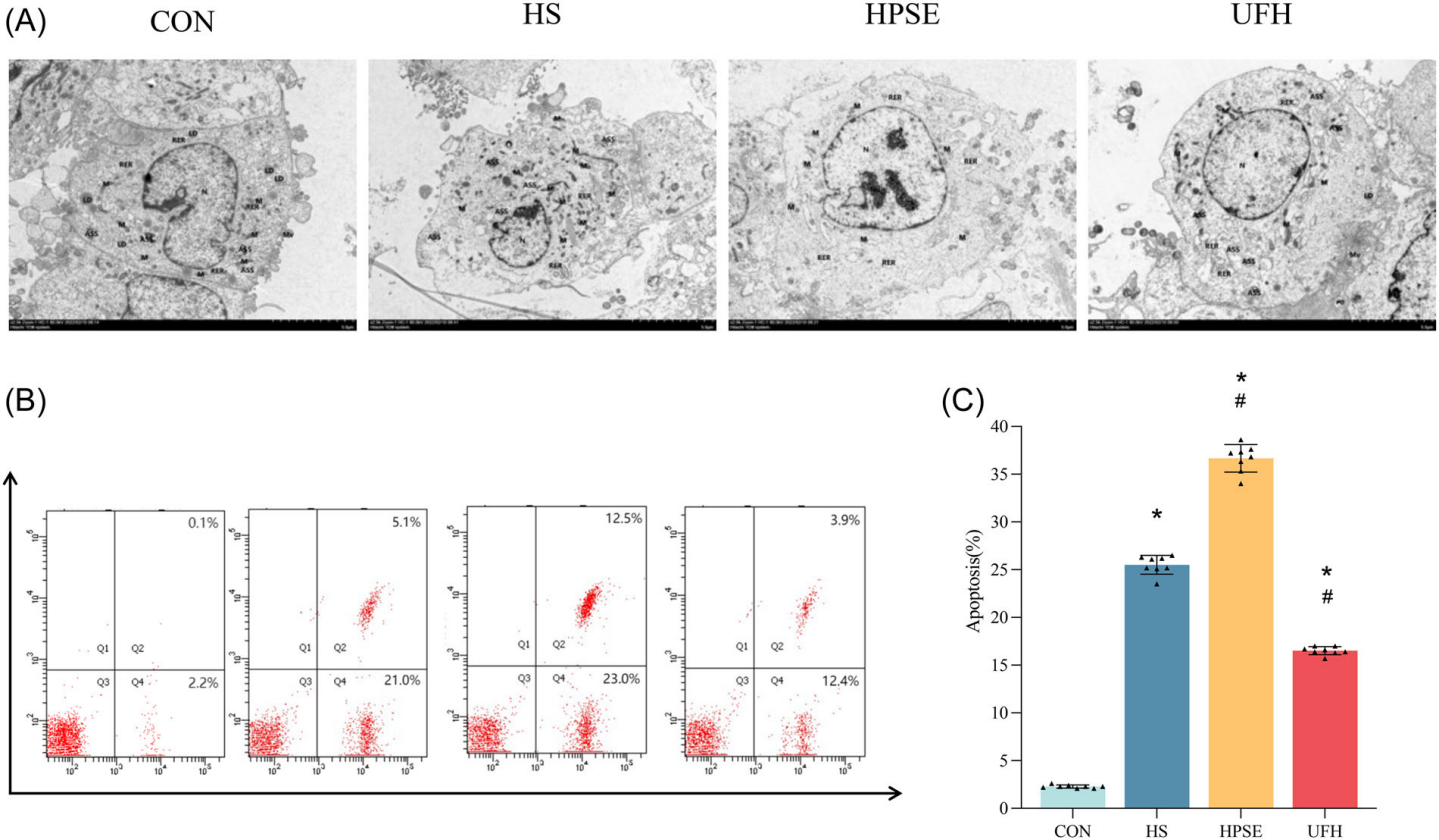
Heat stroke induced cells apoptosis in HPMEC. (A). Representative morphological images of distinct groups in HPMEC using TEM. (B). The apoptotic rates in each group were measured by flow cytometry. (C). Quantitative analysis of the apoptotic rates in distinct groups. *p < .05 versus CON, ^#^ *p* < .05 versus HS; *n* = 8. CON, control; HPMEC, human pulmonary microvascular endothelial cells; HPSE, heparanase; HS, heparansulfate; TEM, transmission electron microscopy; UFH, unfractionated heparin.

The ultrastructure of HPMEC was observed to estimate the effects of heat stress and LPS on HPMEC apoptosis (Figure 2A). Normal cellular structures were observed in the CON group. In model groups, cells generated characteristics of apoptosis such as shrink, and abnormal organelles, including nucleus, mitochondria, and endoplasmic reticulum (Figure 2A). Apoptosis of HPMEC in the HPSE group was more obvious, but cells assigned to UFH were milder than the HS + LPS group (Figure 2A). These results suggested that an altered structure in HPMEC was a result of stimulation of heat stress and LPS.

Evidence suggested that heat stress may lead to cellular apoptosis,^22^ we next analyzed HPMEC activities through flow cytometry using AnnexinV‐FITC/PI staining. As expected, the assignment of HPMEC with heat stress and LPS increased apoptotic rates in both HPSE and UFH treatment, compared with the CON group (*p* < .05) (Figure 2B,C). This increase is more pronounced in the HPSE group (*p* < .05) (Figure 2B,C), suggesting the potential effects of damaged glycocalyx on HPMEC apoptosis.

### 3.3 Heat stress and LPS activated vWF, ET‐1, E‐selectin, VCAM‐1, and occludin released in HPMEC

**Figure 3:**
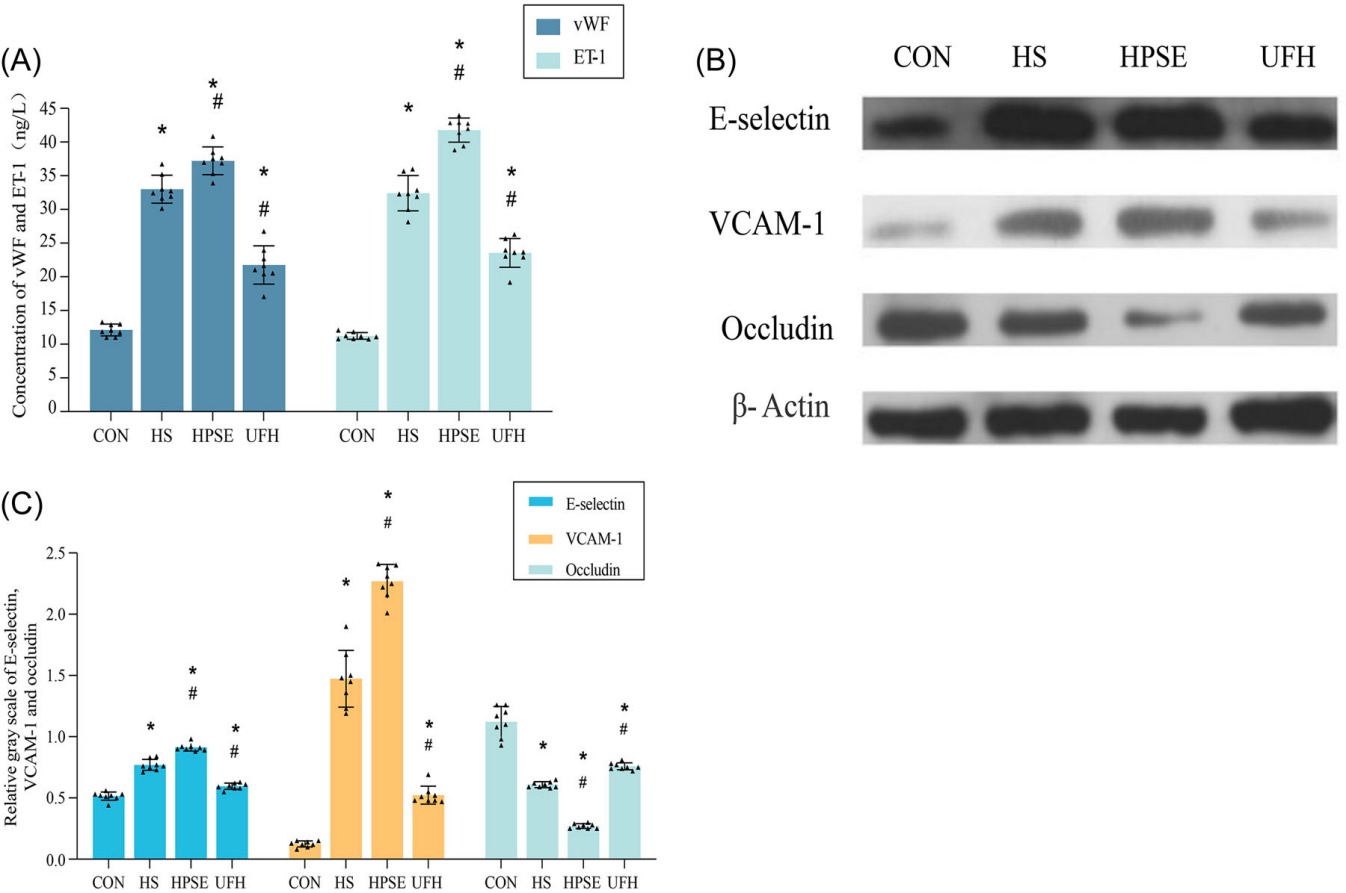
Heat stroke changed expression of coagulation enhancers (vWF and ET‐1), adhesion proteins, and tight proteins. (A). Concentrations of vWF and ET‐1 were performed using ELISA. (B). Western blot was performed to detect expression of E‐selectin, VCAM‐1, and occludin, and quantification of the proteins. (C). Concentrations of E‐selectin, VCAM‐1, and Occludin. The data are presented as mean values ± SD for independent experiments. *p < .05 versus CON, ^#^ *p* < .05 versus HS; *n* = 8. CON, control; ELISA, enzyme‐linked immunosorbent assay; ET‐1, endothelin‐1; HPSE, heparanase; HS, heparansulfate; UFH, unfractionated heparin; VCAM‐1, vascular cell adhesion molecule‐1; vWF, Von Willebrand factor.

To further investigate the effect of heat stroke on the function of coagulation factors, the expression of vWF and ET‐1 was assessed under stimulation of heat stress and LPS. Exposure to stimulation of heat stress and LPS resulted in a statistically significant increase in the expression of vWF and ET‐1 (*p* < .05) (Figure 3A). HPSE treatment significantly increased the generation of vWF and ET‐1, while UFH treatment significantly decreased them in comparison with HS + LPS treatment alone (*p* < .05). These results indicated that heat stress and LPS directly affect glycocalyx degradation as a potential mechanism to induce a clotting state in HPMEC.

Given previous studies indicating the importance of VCAM‐1 and E‐selectin in forecasting cellular inflammation^23^ and the critical role of Occludin in preventing endothelial leakage,^24^, ^25^ in this study, we also aimed to examine the expression of VCAM‐1, E‐selectin, and Occludin. Our results showed that heat stress and LPS significantly enhanced the expression of VCAM‐1 and E‐selectin but attenuated Occludin expression in comparison with the CON group (*p* < .05) (Figure 3B,C). Compared with HS + LPS treatment alone, HPSE treatment significantly upregulated the production of VCAM‐1 and E‐selectin, but downregulated Occludin expression. This data suggested that heat stress and LPS destructed the tight junctions between endothelial cells and promoted the adhesion of inflammatory cells to endothelium, aggravating endothelial leakage in HPMEC.

### 3.4 Degradation of EGCX potentially activated oxidative stress injuries in HPMEC

**Figure 4:**
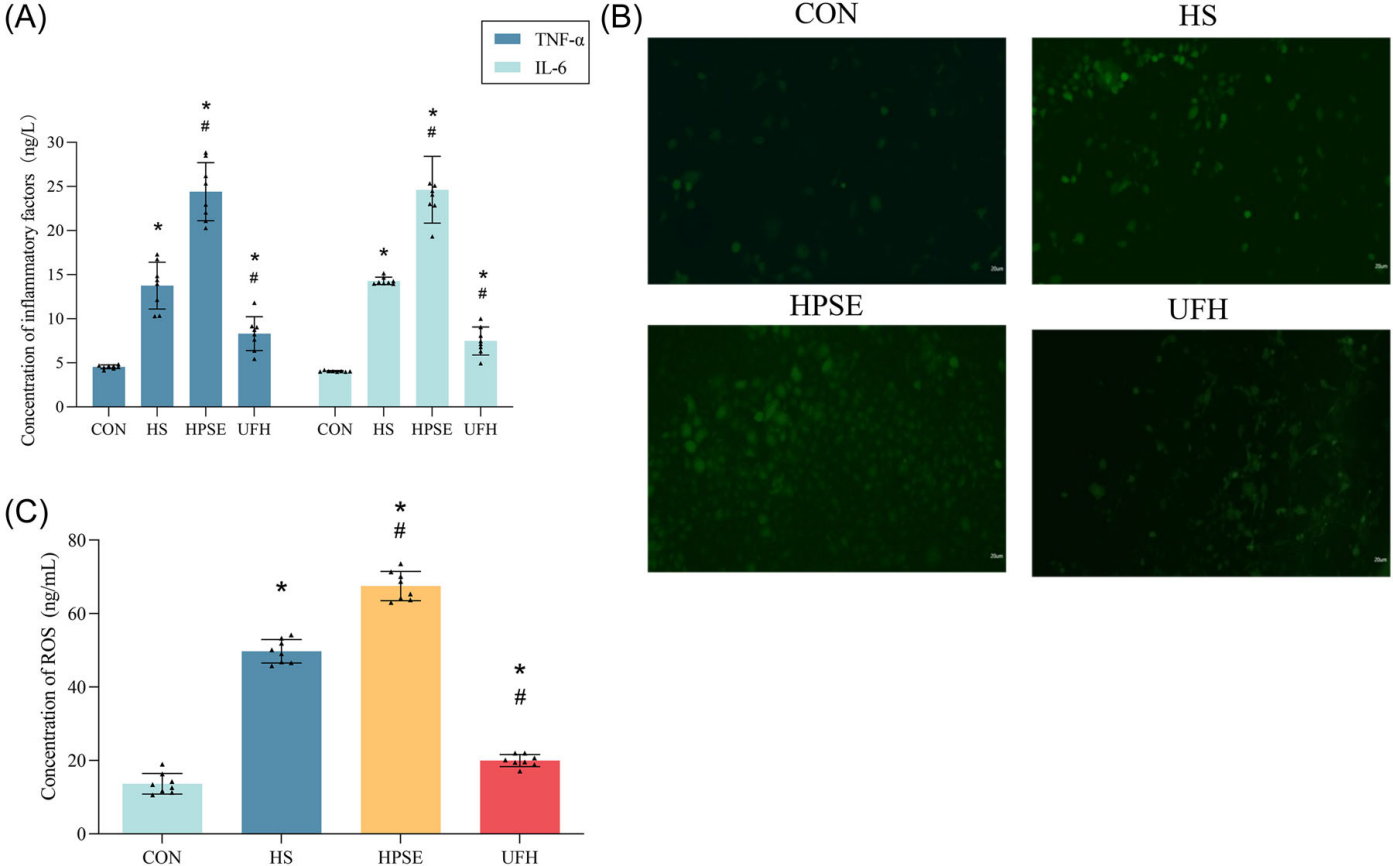
Heat stroke exacerbated the inflammatory state and oxidative stress levels. (A). ELISA was performed to detect concentration of TNF and IL‐6. (B). The level of intracellular ROS was measured with a DCFH‐DA. *p < .05 versus CON, ^#^ *p* < .05 versus HS; *n* = 8. CON, control; ELISA, enzyme‐linked immunosorbent assay; HPSE, heparanase; HS, heparansulfate; IL‐6, interleukin‐6; TNF, tumor necrosis factor; UFH, unfractionated heparin.

TNF‐α and IL‐6 were the classical proinflammation cytokines to accessing inflammatory activities and prognostic outcomes. We, therefore, evaluated the expression of TNF‐α and IL‐6 in treated cells. Our results found that heat stress and LPS significantly increased the production of TNF‐α and IL‐6 in HS, HPSE, and UFH groups, compared with the CON group (*p* < .05) (Figure 4A). This upregulation was more pronounced in cells treated with HPSE but more modest in those treated with UFH in comparison with those treated with heat stress and LPS alone (*p* < .05) (Figure 4A). These results indicated that heat stress and LPS promoted inflammatory activities of HPMEC by an EGCX‐degraded mechanism.

Our previous study demonstrated that generation of ROS was the critical mediator in heat stress‐induced apoptosis,^26^ which was also known to be a classical marker related to oxidative stress in the tissue. We, thus, detected the changes of ROS level in HPMEC by DCFH‐DA marked with green fluorescence, which produces enhanced fluorescence when cells generate ROS. Heat stress and LPS significantly increased the intracellular ROS levels in HPMEC in comparison with the CON group (Figure 4B). In addition, the ROS level was significantly higher in HPSE‐treated cells but was lower in UFH‐treated cells than HS + LPS‐treated cells (*p* < .05) (Figure 4B). These results suggested that oxidative stress of HPMEC is a result of damaged EGCX induced by heat stroke.

## 4 DISCUSSIONS

In this study, the features of vascular EGCX under stimulation of heat stress combined with LPS confirmed vascular inflammatory injury and coagulation disorder. This study showed that heat stress and LPS triggered EGCX degradation and further induced oxidative damage and apoptosis of HPMEC, which impaired its ability to resist inflammatory injury and protecting vascular permeability.

Increase in rectal core temperature induced by heat stress worsened intestinal lesions in mice, leading to a dislocation of endotoxin, and increasing in the level of endotoxin.^14^, ^15^ That was believed as a key link in the pathogenesis of severe heat stroke.

HSPG was a major element of glycocalyx, which played important roles in endothelial cell homeostatic signals based on their unique structures and interactions with both the intra‐ and extracellular environments. Decreased HSPG prompted degradation of EGCX and vascular disease.^20^ In this study, a lower level of HSPG was detected in HPMEC under heat stress and LPS compared with controls, which was consistent with the previous study.^27^ As the major mammalian enzyme of degrading vascular EGCX, HPSE was believed to aggravate the shedding of EGCX.^28^, ^29^ Conversely, UFH was known to consolidate the structure and strengthen the function of glycocalyx via noncovalent binding.^30^ Thus, UFH was believed to attenuate the degradation of EGCX.

High concentrations of serum HS and SDC‐1 were used as principal markers of EGCX shedding, as demonstrated in previous studies.^27^, ^31^ In this present study, treatment with heat stress and LPS significantly increased the concentration of serum HS and SDC‐1, compared with the CON group. Preconditioning with HPSE enhanced EGCX damage, whereas UFH attenuated it. These results reconfirmed that heat stroke triggered the degradation of EGCX.

Earlier studies demonstrated that heat stress contributed to injury and apoptosis of endothelial cells by activation of inflammation, which played an important role in organ injury secondary to heat stroke.^32^ Given this timeline, our results showed that heat stress and LPS‐induced endothelial cells apoptosis morphologically, which were more significant in HPSE‐treated cells. A recent study found that endothelium apoptosis may be induced and aggravated by EGCX injury. Collectively, our results suggested that the activation of endothelium apoptosis may be highly associated with EGCX damage as a result of heat stroke.

Inflammation and coagulation played a critical role in the pathophysiological basis of tissues/organs damage secondary to heat stroke.^33^ Moreover, abnormal structure and function of endothelium exacerbated inflammation through increasing vascular permeability and promoting adhesion of inflammatory cells, as well as producing massive inflammatory mediators and procoagulants. However, the development of inflammation and coagulation after heat stroke‐induced glycocalyx injury was still to be elucidated. Given this association, it was essential to explore the relationship between inflammation and glycocalyx damage.

TNF‐α was known to promote inflammation via upregulating the expression of adhesion factors on the surface of the endothelium, such as VCAM‐1 and ICAM‐1.^34^ IL‐6 induced intracellular cascade signal transduction by binding to the IL‐6 receptor, which amplified the effect of TNF‐α and promoted the development of inflammation. promoted the development of inflammation.^35^, ^36^ Moreover, IL‐6 played a strong roles in promoting inflammation in the pathogenesis of MODS secondary to heat stroke.^36^ vWF and ET‐1 were associated with the function of promoting coagulation.^37^ Endothelium was effective in constricting blood vessels via releasing ET‐1.^37^ In addition, endothelium damage was mediated by inflammatory factors, which contributed to enlarging endothelial space and further aggravating inflammatory response.^38^ These may explain the upregulation of TNF‐∂, IL‐6, vWF, and ET‐1 in injured glycocalyx treated with heat stress and LPS, especially in HPSE‐treated cells. Collectively, these results might imply that heat stroke‐induced glycocalyx degradation enhanced the activation of inflammation and coagulation.

E‐selectin and VCAM‐1 were the important symbols to reflect injury of EGCX and endothelium, which also played critical roles in the spread of inflammation via mediating the adhesion between leukocyte and inflammatory cells.^23^, ^39^, ^40^ In our results, increased expressions of VCAM‐1 and E‐selectin were significantly observed in endothelium treated with heat stress and LPS, especially in cells suffering from HPSE. These findings were consistent with those that revealed a significant upregulation of E‐selectin in injured vascular EGCX,^41^, ^42^ which confirmed a dysfunction of endothelial cells inhibiting inflammation. Occludin was effective in preventing endothelium from leakage, localized in the tight junctions between cellular preferentially.^24^, ^25^ In the present study, western blot analysis revealed that the expression of occludin was aggravated by injured EGCX in HPMEC. These data suggested that heat stroke possibly triggered EGCX damage, which resulted in endothelial tight junction injury.

Decreased occludin may be associated with widening gaps between endothelial cells, which aggravated the vascular leakage. Our data indicated that heat stroke‐induced glycocalyx damage enhanced adhesion protein expression, and further led to pathophysiological changes, including increased vascular permeability and leukocyte adhesion.^43^ It is consequently hypothesized that EGCX damage may be the key pathophysiological basis of dysfunction of endothelium and inflammatory damages induced by heat stroke.

ROS was also associated with the degradation of EGCX, widely involved in signaling transduction and the life process of cells. Excessive ROS may lead to diseases by inducing oxidative stress in mitochondria.^44^ Further studies showed that under heat stroke, increased inflammatory factors mediated iNOS to generate NO, and further generated excessive ROS, which was associated with heat stroke‐induced ALI.^45^ At the same time, heat stress directly induces an increase in intracellular ROS and leads to cell apoptosis.^46^ In the present study, the relationship between intracellular oxidative stress and heat stroke was confirmed by an overproduction of ROS under heat stress, which may be associated with the extent of severity of glycocalyx damage.

Release of ROS may trigger EGCX degradation when exposed to generation of TNF‐α and endotoxin, as well as inflammatory states, including sepsis.^47^ ROS produced during sepsis initiated the feed‐forward cascade of inflammation and glycocalyx degradation.^43^, ^48^ EGCX was an important site of action of circulating ROS and cytokines generated by oxidative stress, acting a protective role in vascular endothelial cells damaged by ROS. Hence, degradation of EGCX increased the risk that vascular endothelium was damaged by ROS, leading to endothelial dysfunction.^49^

As the most abundant molecular chaperone proteins, heat shock proteins (HSPs) expressed under physiological conditions, and increased in expression when cells were exposed to high temperature or hypoxia.^50^ Previous studies have reported that heat‐triggered HSP70 expression protected cells from various types of lethal damage. HSP70 was the most sensitive to heat stress, which protected cells from heat stress by inhibiting inflammation and oxidative damage.^51^ HSPs were considered immune modulators in many bodily fluids due to their role in inhibiting increased expression levels of pro‐inflammatory cytokines, including IL‐6 and IL‐8.^52^, ^53^ On the association between the protective effects of HSPs and EGCX damage‐induced inflammation and oxidative stress, very little research has been conducted. Multi‐omics analysis is needed to explore the roles of HSPs in the pathogenicity of heat stress and EGCX damage in our further studies.

There was a limitation in our study. The mechanism of EGCX contributing to inflammatory and oxidative responses related to heat stroke was unclear. Future studies are needed to explore the potential signal pathways and mechanism of dysfunction of vascular endothelium induced by EGCX degradation under heat stroke.

## 5 CONCLUSIONS

In conclusion, our study provides evidence that degradation of endothelial glycocalyx induced by heat stress and LPS causes endothelium cell apoptosis in HPMEC, which leads to increased generation of inflammatory cytokines and oxidative response, thereby contributing to aggravating inflammation and destroying the vascular permeability.

## AUTHOR CONTRIBUTIONS

**Jiadi Chen**: Data curation; formal analysis; project administration; writing—original draft; writing—review & editing. **Chengjia Ding**: Data curation; formal analysis; software. **Jingjing Cao**: Data curation; formal analysis; software. **Huasheng Tong**: Funding acquisition; methodology; project administration; writing—review & editing. **Yi Chen**: Conceptualization; funding acquisition; methodology; project administration; writing—review & editing.

## CONFLICT OF INTEREST STATEMENT

The authors declare no conflict of interest.

## Data Availability

The data that support the findings of this study are available from the corresponding author upon reasonable request.
